# Pathological mandibular fracture: A severe complication of periimplantitis

**DOI:** 10.4317/jced.52305

**Published:** 2015-04-01

**Authors:** Luis Naval-Gías, Francisco Rodriguez-Campo, Beatriz Naval-Parra, Jesús Sastre-Pérez

**Affiliations:** 1 PhD, DMD. Maxillofacial Surgeon. Oral and Maxillofacial Surgery Department. Hospital Universitario La Princesa. Madrid. Spain; 2 MD. Maxillofacial Surgeon. Oral and Maxillofacial Surgery Department. Hospital Universitario La Princesa. Madrid. Spain; 3 DDS. Private practice Madrid Spain; 4 MD. Maxillofacial Surgeon. Oral and Maxillofacial Surgery Department. Hospital Universitario La Princesa. Madrid. Spain

## Abstract

Nowadays, dental implant treatment is a very common option for patients even in medical compromised conditons. Some complications related to them have been described. Periimplantitis (PI) is one of the biggest concerns complications of these kind of treatments, probably has a multifactorial aethiology. Usually the consequences of PI are the loss of the implants and prostheses, expenses of money and time for dentists and patients. Very often PI implies the necesity of repeating the treatment . Pathological mandibular fracture due to PI is a severe but infrequent complication after dental implant treatment, especially after PI. In this study we present three cases of mandibular pathologic fractures among patients with different medical and dental records but similar management: two of them had been treated years ago of oral squamous cell carcinoma with surgery and radiotherapy, the other patient received oral bisphosphonates for osteoporosis some years after implantation. We analized the causes, consequences and posible prevention of these fractures as well as the special features of this kind of mandibular fractures and the different existing treatments.

** Key words:**Periimplantitis, pathological mandibular fracture, mandibular atrophy, bicortical implants.

## Introduction

Osseointegrated dental implant for restoring oral aesthetics and function in atrophic mandible is highly successful and predictible procedure even in medical compromised patients. However, this treatment is not exempt of complications(1). Side effects like wound infection, hemorrhage, neurosensory disturbance, prosthetic problems, periimplant mucositis or periimplantitis and mandibular fracture have been reported (2-6). Mandibular fracture associated with dental implants treatment is related with dental implant instalation procedures, after inferior alveolar nerve transposition technique or mandibular distraction before implant procedure. Very few cases have been reported relating periimplantitis. The authors report three cases of pathological mandibular fracture in patients presenting severe periimplant disease.

## Case Report

-CASE 1

A 72 years-old woman presented history of oral squamous cell carcinoma located in anterior floor of the mouth. 12 years earlier she underwent surgery, including marginal mandibulectomy, neck disection, and reconstruction using a radial fasciocutaneous free flap. She also received 70 Gy of postoperative radiotherapy. Four years later, an anterior iliac crest bone grafting was performed to gain mandibular vertical dimension. Five dental implants were inserted in the interforaminal area of the mandible and four in the maxilla (external hex connection). The patient’s quality of life improved significally after the implant supported rehabilitation.

**Figure 1:**
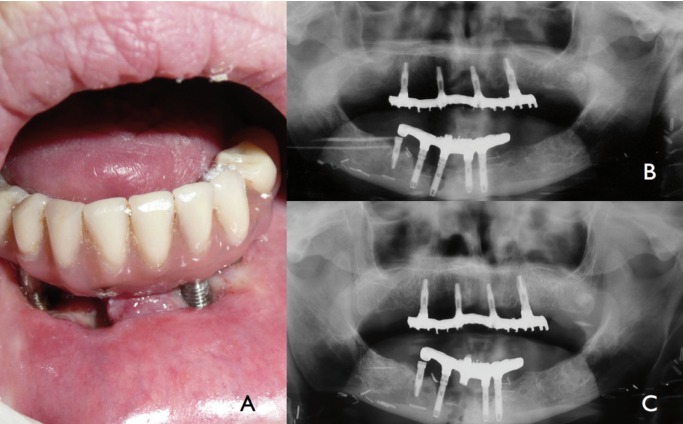
Case 1: A) Intraoral view of clinical periimplant disease implant body exposure. B) Advanced periimplant disease in orthopantomography around three mandibular implants. Maxillary fixed prothesis supported by four implants without signs of PI. C) Central implant removed and prothesis fixed again.

Eleven years after the implant loading, she complained about discomfort and gingival swelling around the chin. Clinical examination revealed mucositis and purulent exudate from two implant sites. The panoramic X-ray showed a significant loss of bone around three anterior mandibular implants (Fig. 1).

The prosthesis was temporarily removed with one lost implant. A few days later, she reported increased pain and local crepitation. X-Ray confirmed a mandibular midline fracture at the site of the removed dental implant.

**Figure 2:**
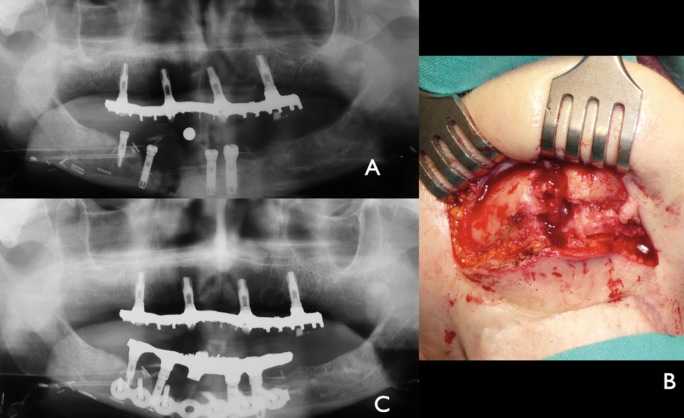
Case 1: A) Anterior mandibular fracture at the site of one of the implants with PI. B) Extraoral approach to expose mandibular fracture. C) Orthopantomography postoperative showing fracture reduction and stabilization by titanium reconstruction plate.

-Treatment: An open reduction and internal fixation (ORIF) under general anesthesia was performed by extraoral approach. The prothesis was used to guide the reduction and fixation of the fracture by means of a titanium reconstruction plate (Fig. 2). At this time the patient was under treatment for another second primary tumor in the rectus. Bone graft was not considered because of the infected tissue. The patient was discharged 3 days postoperatory uneventfully wearing her prosthesis adjusted to the new situation. A submandibular fistula was observed at the implant failed site). Initially, the patient and family declined any further surgeries and remained stable for two years. No periimplant bone loss was observed around maxillary implants. Because of the persistent fistula we performed an osteocutaneous fibula flap. The patient died because of lung metastases few months after this last surgery.

-CASE 2

A 63-year-old-woman was referred to our Department complaining of swelling and pain located in the chin added to chewing difficulty. The patient had been wearing a complete inferior fixed prosthesis for the last five years, supported by 5 osseointegrated dental implants of internal connection and swicht plattform. The patient medical records included general osteoporosis treated with oral bisphosphonates 3 years after implantation. A conservative treatment was recomended by her dentist. She was told she had a bone related osteonecrosis of the jaw (BRONJ).

Intraoral examination revealed gingival swelling and purulent discharge around implants with movility between both sides of the mandible. No bone exposure was evident.

**Figure 3:**
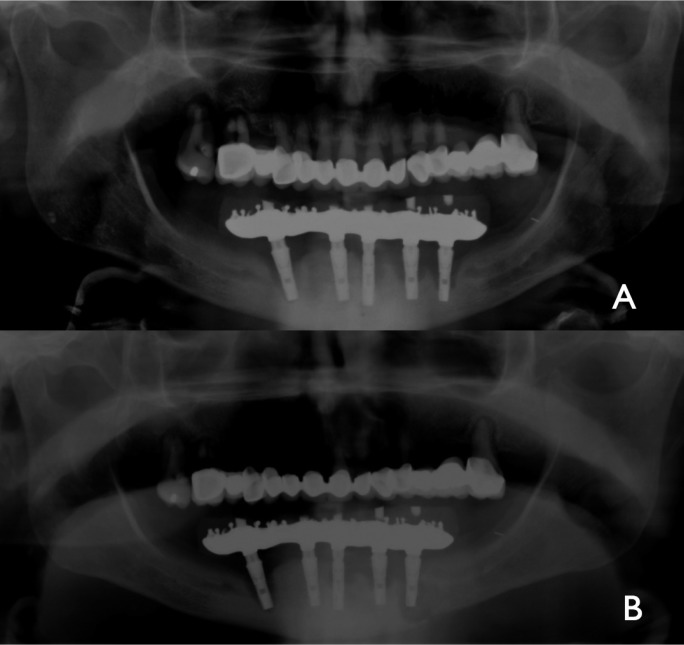
Case 2: A) showing PI around most of the implants. B) Panoramic radiography revealing pathological mandibular fracture around left distal implant.

The panoramic X-ray revealed a left mandibular fracture around distal implant and bone loss around most of the fixations (Fig. 3). The prosthese was removed with the two distal implants. Nevertheless the transmucosal abutments the design of the prostheses clearly favored plaque acumulation.

-Treatment

**Figure 4:**
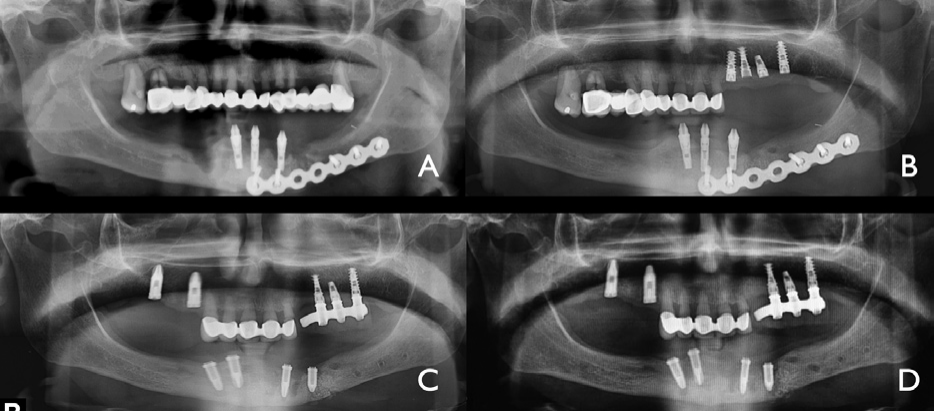
Case 2: A) Panoramic radiography showing rigid fixation using a reconstruction plate. B) Eight months later, a good healing of mandibular fracture is observed. C) The titanum plate is removed. New mandibular and maxillary implants are placed. D) One year later, mild periimplant bone loss is observed around implants.

Under general anesthetics, through an extraoral approach an ORIF with a titanium reconstruction plate, filling the bone defect with xenograft was performed. The patient was discharged uneventually three days after surgery. Six months later, the remanent implants and reconstruction plate were intraorally removed with contratorque device (Neobiotech®). At this time good bone consolidation was observed and 4 new mandibular implants were placed (Fig. 4). One year later, some bone loss was observed around these implants. New implants are not yet connected because the patient refused an overdenture.

-CASE 3

**Figure 5:**
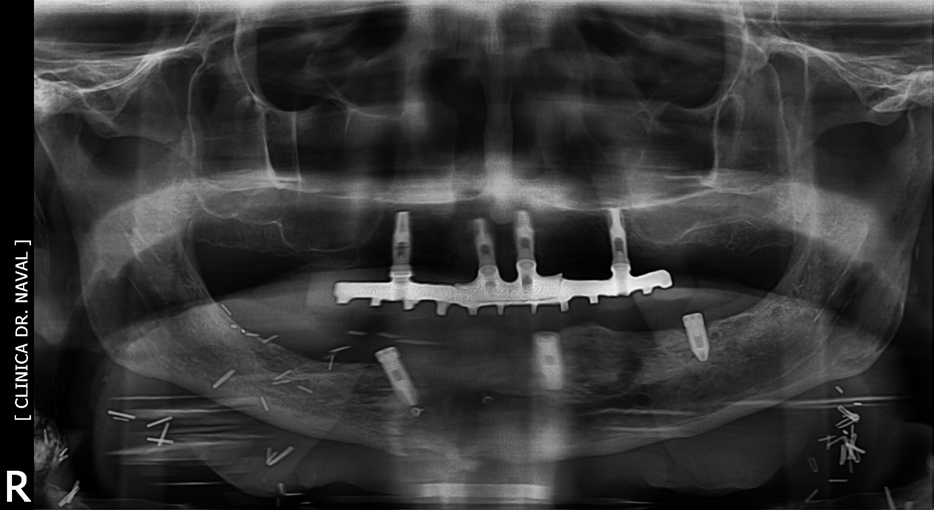
Case 3: Second episode of PI at the same site, lead to a pathologycal mandibular fracture. Maxillary implants do not reveal any bone loss.

A 72 years old patient, with tobacco and alcohol habits, had been treated, 23 years ago, with surgery and radiotherapy for an squamous cell carcinoma of the anterior floor of the mouth on the rigth side. He received a first dental implant treatment at the mandible as well as the maxilla some years after the oncologyc treatment with a great improvement of his quality of life. Four implats in each arch were used and a hybrid acrilyc fixed denture was built for both arches. Six years after implant placement, he developed PI and lost two implants. At the same place Guided Bone Regeneration (GBR) protocol was performed with both auto-logous shavings from a disposable scraper and xenograft and a collagen membrane fixed by tacks. Seven months later the treat-ment was performed again adding two more implants. The patient continued with addiction to tobacco and alcohol, in fact he developped a hepatyc cirrosis. When the patient came to us, four years later a new episode of PI was observed around two implants at the same place. The prosthese was removed with two implants and 2 months after he presented chin pain, crepitation and orocutaneous fistula. The Panorex and Cone Beam Computed Tomography (CBCT) showed a mandibular fracture (Fig 5). The patient underwent surgery: ORIF. He also received a course of Hyperbaric Oxygen therapy. Now he is asyntomatic concerning the oral cavity but waiting for bone regeneration because of hepatyc condition.

## Discussion

Periimplant disease is a multifactorial infectious disease related with several factors related to the patient like previous periodonti-tis, diabetes, radiotherapy, presence of attached gingiva, poor hygiene, tobacco habit, etc. or the implant design (rough surfaces, plattform type, etc. and the prostheses (3-6). Mucositis affect periimplant soft tissue causing gingival swelling, bleeding on probing or supuration. These changes are reversible when proper local treatment is applied. Whereas that PI implies also changes in crestal bone level is more difficult to treat (3-5). Some authors consider early explantation as an alternative to prevent bone loss (5,6).

In our serie of three cases, some factors were the promotor of PI like radiotherapy in two cases, tobacco in the other and history of periodontal disease in all of them. Two patients wore external hex implants treated with acid in one case and RBM (reabsorbable blast media) in other and the thirth patient wore implants with “plattform swichting”. All three of the patients had more than one implant affected by PI.

Some ethiologyc factors are related to pathological mandibular fractures, including PI which causes loss of supporting bone and structural mandibular weakness and, occasionally, mandibular fracture (7-11). This could be originated by mild trauma or routine chewing specially when implants are installed bicortically (11,12).

It has been proven how radiotheraphy (RT) for cancer treatment causes endarteritis and hipoxia (13,14). The irradiated tissues are more susceptible to local damage. Even several years following RT and ussually associated to a local trauma (dental extraction, implant placement….) an osteoradionecrosis could be developed. This process could be also responsible of osseointegration failure in irradiated patients . Other studies concluded that dental implant osteointegration is possible and stable in irradiated patients, but that they must be exhaustively informed about complications (13-14).

Bisphosphonate (BP) induced osteonecrosis BRONJ is other factor related to dental implant complications and mandibular pathologyc fracture. The American Association of Oral and Maxillofacial Surgeons guideline (15) contraindicated dental implant placement in patients who had undergone intravenous BP treatment. However, it is posible in patients that received oral BP for less than three years and do not present any added risk factors like intake of corticosteroids. BP treatment seems not to be related to our second case because the BP treatment begun after the implant placement and no bone exposure was observed. Both factors (RT or BP), contribute to alter the structural strenght of the mandible, enabling pathological mandibular fracture. PI could be acting as a trigger factor.

Pathological fractures often occur in atrophic mandibles or edentulous patients. Generally, these fractures follow AO/ASIF principles and are treated via extraoral transcervical approach, wide exposition of focus fracture, open reduction and rigid internal fixation using a reconstruction plate (1,11) This is not the only way to treat mandibular fractures, a wide range of treatment options are possible, depending on the patients characteristics. O´Sullivan (2) reported in 2006 one case of mandibular fracture secondary to osteomyelitis related to a periimplant disease. In this particular case only conservative management, soft diet, antibiotics and oral hygiene regimen were enough to promote bone healing. Almasri (7) in 2012 reported a case of mandibular fracture secondary to periimplantitis treated by means extraoral open reduction, internal fixation (ORIF) and stabilization with a 2.4 mm reconstruction plate, removing the implant. He reported two complications: wound dehiscence and inferior alveolar nerve parestesia that were managed conservatively. In 2009 Chrcanovic (9) reported four cases of pathological fractures, three following periimplantitis and another after inferior alveolar nerve transposition. In all cases, they used a transcervical approach and ORIF with 2.0 miniplates. A complication was presented, a plate fracture related to severe bruxism which was treated using intermaxillary fixation (IMF). Some authors recomend not to fix mandible fracture using ORIF if the fracture is not displaced, just IMF using the patient´s protheses to guide the occlusion and to reduce the fracture focus (1,7,8,11) In the same fashion, we used the patient´s prostheses to improve the reduction of the fragments, specially if there is some lost of tissue like in our cases.

Other authors reported an extraoral approach and ORIF using a compression plate without complications in a case of mandibular fracture following bicortical engagement and wide diameter of dental implants (8).

We prefer the ORIF modality to treat pathological fractures to ensure good stability. In our cases 1 and 3, no bone grafing was initially performed due to an infected bed and bad mucosal closure. A second stage surgery was made in case 2 in order to close the cutaneous fistula.

## Conclusions

Pathological mandibular fractures following periimplant disease are a rare but severe complication after dental implant treatment. There can only be found three papers in literature on these type of complications. Complete disclosure concerning the risks of the implant procedure should be discussed with the patients, especially in very atrofic or risky cases like those treated with radiotherapy. The bicortical installation of the fixtures or the use of large implants improves stability but can lead to a mandibular pathologyc fracture in cases of severe PI. A close patient follow-up is indispensable to early diagnosis and treatment in cases of periimplant disease in atrophic mandibles. In some cases of PI with risk of mandibular fracture, early explantation should be considered using counter torque devices. In none of these patients maxillary PI was observed.
